# Tissue Engineering in Maxillary Bone Defects

**Published:** 2018-01

**Authors:** Azizollah Khodakaram-Tafti, Davood Mehrabani, Hanieh Shaterzadeh-Yazdi, Barbad Zamiri, Mahsa Omidi

**Affiliations:** 1 Department of Pathobiology, School of Veterinary Medicine, Shiraz University, Shiraz, Iran;; 2 Burn and Wound Healing Research Center, Shiraz University of Medical Sciences, Shiraz, Iran;; 3 Comparative and Experimental Medicine Center, Shiraz University of Medical Sciences, Shiraz, Iran;; 4 Department of Craniomaxillofacial Surgery, School of Dentistry, Shiraz University of Medical Sciences, Shiraz, Iran;; 5 Department of Oral and Maxillofacial Radiology, School of Dentistry, Shiraz University of Medical Sciences, Shiraz, Iran

**Keywords:** Fibrin glue, Scaffold, Autologous bone graft, Mandibular defect, Rabbit

## Abstract

**BACKGROUND:**

Restoration of craniofacial bone defects has been a concern for oral and maxillofacial surgeons. In this study, the healing effect of fibrin glue scaffold was compared with autologous bone graft in mandibular defects of rabbit.

**METHODS:**

Bilateral unicortical osteotomy was performed in the diastema region of 10 male Dutch rabbits. The subjects were randomly divided into 2 equal groups. The mandibular defect on the right side was treated with fibrin glue scaffold and the defect on the left side with autologous bone graft provided from iliac crest. After 4 and 8 weeks, five rabbits from each group were sacrificed and the defects were evaluated morphologically, by coronal computed tomography scanning (CT-scan) and by histological examinations.

**RESULTS:**

The healing effect of fibrin glue scaffold and autologous bone graft was similar with appropriate osteogenesis in comparison to the control group.

**CONCLUSION:**

Using fibrin glue can be a non-invasive treatment of choice in mandibular defects and maxillofacial surgeries when compared with autologous bone graft.

## INTRODUCTION

Restoration of craniofacial bone defects resulting from congenital faults, injuries or tumor amputation, has been a concern for oral and maxillofacial surgeons and orthopedic surgeons.^1^ As new bone formation happens via osteogenesis, osteoconduction or osteoinduction processes,^2^ autologous bone graft from the iliac crest has been used to provide all the necessary elements for bone restoration and has been as a gold standard in treatment of bone defects.^3^ However, problems such as providing another surgical site, limited amounts of available bone graft in children and seniors, possibility of bone graft particle moving during their placement,^4^ nerve injury, donor site morbidity and cosmetic disadvantages reported during autologous bone grafts.^5^^-^^7^

Regenerative medicine including two different strategies of cell therapy and tissue engineering has opened a new window in orthopedic surgeries. Since cell therapy alone may not be enough for restoration of large bone defects, now extensive studies have focused on tissue engineering together with stem cells to support osteogenic potency, a suitable scaffold and growth factors for healing and reconstruction of bone defects.^8^^-^^10^

Fibrin glue is consisted of 2 major components of fibrinogen and thrombin and has been considered as an attractive scaffold in tissue engineering. It can improve cell migration and proliferation. Also, it has growth factors which can stimulate and promote tissue reconstruction.^11^ Compared to commercial fibrins, autologous fibrin scaffolds are excellent candidates for tissue engineering because of being faster, cheaper and in possibility to produce in larger quantities and also with fewer viral or prion risk of infection.^11^^,^^12^ Computed tomography (CT) scanning was shown to be able to provide an easy, quick and precise survey in maxillofacial injuries, whereas conventional radiologic techniques cannot usually display the details of comminutions seen during surgeries of multiple injuries.^13^ Therefore, this study was undertaken to compare the healing and regenerative effects of autologous fibrin glue, and autologous bone graft in mandibular segmental cortical defects in rabbit model assessed by histological examinations.

## MATERIALS AND METHODS

Ten mature male Dutch white rabbits aged 6 months with a mean weight of 2.5 kg were provided from the Laboratory Animal Center of Shiraz University of Medical Sciences, Shiraz, Iran. They were housed, treated and euthanized based on guidelines of *Animal Care Committee *of *Iran Veterinary Organization.* The study was also approved in the institution ethics committee.

Before surgery, 25 ml blood was drawn from the heart of each rabbit and transferred to a syringe containing citrate dextrose phosphate (CPD). The blood was centrifuged and the plasma was separated from red blood cells and buffy coat. To prepare autologous fibrin glue from the obtained plasma, Thorn *et al.*^14^ method was modified. Briefly, a part of the obtained plasma was separated to prepare thrombin and the remaining was used to provide fibrinogen. Extraction of the fibrinogen was done by adding of tranexamic acid and ethanol to the plasma and incubation in the water bath for 20 to 30 minutes, respectively. 

**Fig. 1:**
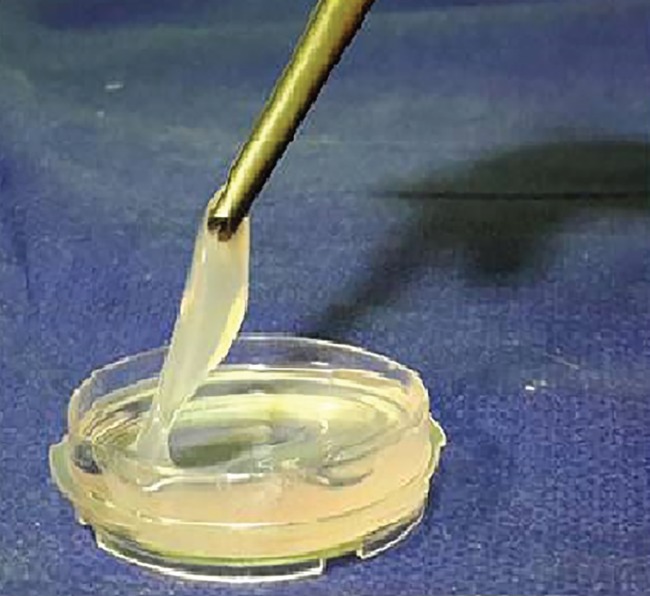
A gel-like fibrin glue formation after mixing of fibrinogen and thrombin.

Finally, after centrifuging of the precipitated fibrinogen, the supernatant removed and the precipitate was dissolved in NaCl. To prepare the thrombin, briefly, citric acid was added to the remaining plasma. After centrifuging of this mixture and discarding of the supernatant, the precipitate was dissolved in CaCl_2_. To reach to a neutral pH, NaHCO_3_ was added. After 20-30 minutes, liquid thrombin was removed and after mixing of fibrinogen and thrombin, a gel- like fibrin glue was formed (Figure 1). 

In rabbits, a total of 40 mandibular cortical defects were created bilaterally. The subjects were randomly divided to 2 equal groups. The mandibular defect on the right and left sides were filled with fibrin glue (without stem cells) and autologous bone graft (from iliac crest), respectively. The rabbits underwent general anesthesia using 44 mg/kg of 10% ketamine (Alfasan, Woerden-Netherlands) and 10 mg/kg of 2% xylazine (Rompun, Bayer AG, Leverkusen) intramuscularly. The skin around the ventral region of mandible and neck areas were shaved and disinfected.

After creation of an incision in the skin and dissection of the muscles, bilateral 1.5×0.5 cm unicortical osteotomies were performed into the mandibular diastema space between the incisor and first molar teeth with dental drill under copious irrigation. For filling of the defect with fibrin glue, a fibrin clot of fibrinogen and thrombin were added. For covering the defect with autologous bone graft, harvesting of bone particles was conducted from iliac crest during the same operation. Finally, the incision was sutured in layers and piperacillin (22000 IU/Kg) and flunexin meglumine (0.15 mg/Kg) were administered as antibiotic and analgesic agents for three days. After 4 and 8 weeks, five rabbits from each group were sacrificed and the defects were evaluated by gross, coronal CT scanning and histological examinations.

After euthanasia, the surrounding tissues of the defective site in each mandible were cleaned and were evaluated grossly and Images were captured using a digital camera. To compare bone reconstruction, 3 mandibles from each treatment and control groups were randomly assessed by a spiral CT scan apparatus (Light Speed Ultra16; General Electric, Milwaukee, WI, USA) at 80 kV and 3 mA. 

For histological evaluation, the bone samples were fixed in 10% buffered formalin, and transferred in 15% nitric acid for decalcification. The calcified samples were routinely processed and embedded in paraffin blocks. Subsequently, 5 µm thick sections were cut and stained by hematoxylin-eosin. The specimens were evaluated under a light microscope (CX21; Olympus, Japan). For a semi-quantitative histological evaluation, a modified grading system based on the Emery’s *et al*.’s method^15^ was applied as follows: 

Grade 0: when the gap was empty, Grade 1: if the gap was ﬁlled with ﬁbrous connective tissue only, Grade 2: if the gap was ﬁlled with ﬁbrous tissue more than ﬁbrous-immaure bone tissue, Grade 3: when ﬁbrous-immaure bone tissue was more than ﬁbrous tissue, Grade 4: when the gap was covered with ﬁbrous-immaure bone, Grade 5: ﬁbrous-immaure bone was more than mature bone, Grade 6: mature bone was more than ﬁbrous-immaure bone tissue and Grade 7: if the gap was only completed with mature bone. 

Also, another scoring system was used to evaluate presence and width of cortical bone as described by An and Friedman^16^ as follows: Grade 0: absence of cortical bone, Grade 1: cortical bone surface <50% of the defect, Grade 2: cortical bone surface > 50% of the defect and Grade 3: complete cortical bone. The thickness of the newly formed cortical bone bridge was measured by stereological equipment including a microscope (Nicon, Japan) with an electronic microcator (Heidenhain MT-25, Germany) connected to a computer in stereology and histomorphometry Research Center of Shiraz University of Medical Sciences, Shiraz, Iran. The stereological probes contained point nets and counting frameworks imposed on images of the tissue sections. The newly formed cortical bone thickness was established in 10 different regions randomly and the mean was considered as thickness of cortical bony bridge.

Quantitative data were presented as the mean±standard deviation. Statistical analysis of the histological results between different groups was done using non-parametric Kruskal–Wallis test. When *P* values were less than 0.05, pair wise group comparisons were performed by Mann–Whitney U test (SPSS software, version 16.0 for windows, Chicago, IL, USA).

## RESULTS

**Fig. 2:**
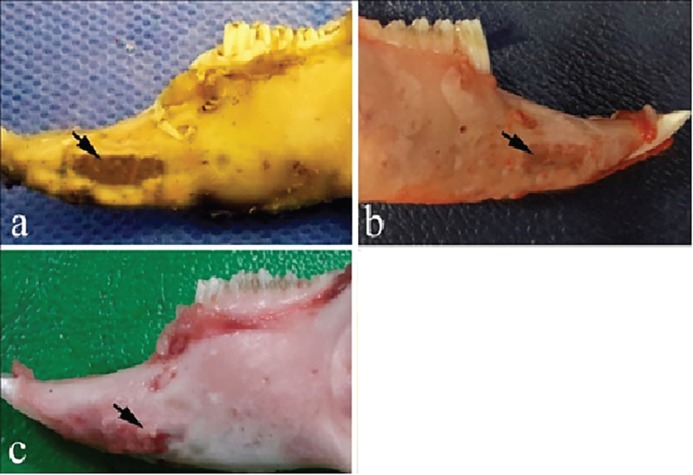
Macroscopic evaluation of the bone samples 4 weeks after surgery. **a)** the control defect, **b)** the defect was filled by fibrin glue alone, **c)** the defect was filled by autologous bone graft.

**Fig. 3:**
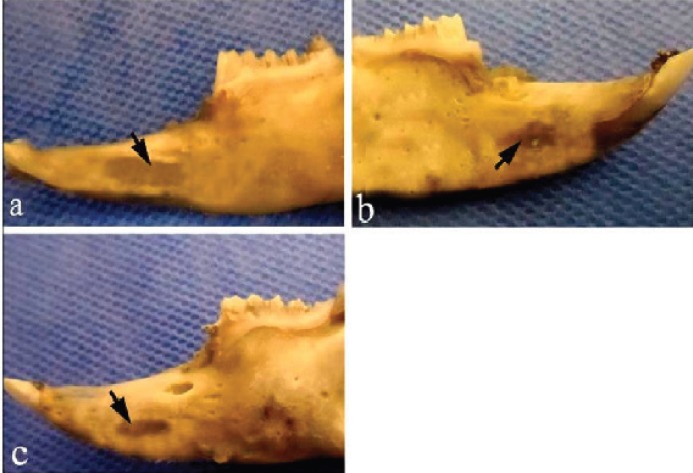
Macroscopic evaluation of the bone samples 8 weeks after surgery. **a)** the control defect, **b)** the defect was filled by fibrin glue alone, **c)** the defect was filled by autologous bone graft. Arrows denote the defect area.

Macroscopically, after 4 weeks in the control group, the defects were almost covered with granulation tissue, while the margins of the defects were still recognizable in the defects filled with fibrin glue alone and autologous bone graft (Figure 2). After 8 weeks, a new cortical bone formation was detected in the control defects without thickening, while the margins of the defects were recognizable when fibrin glue alone or autologous bone graft was used to fill the defects (Figure 3).

**Fig. 4:**
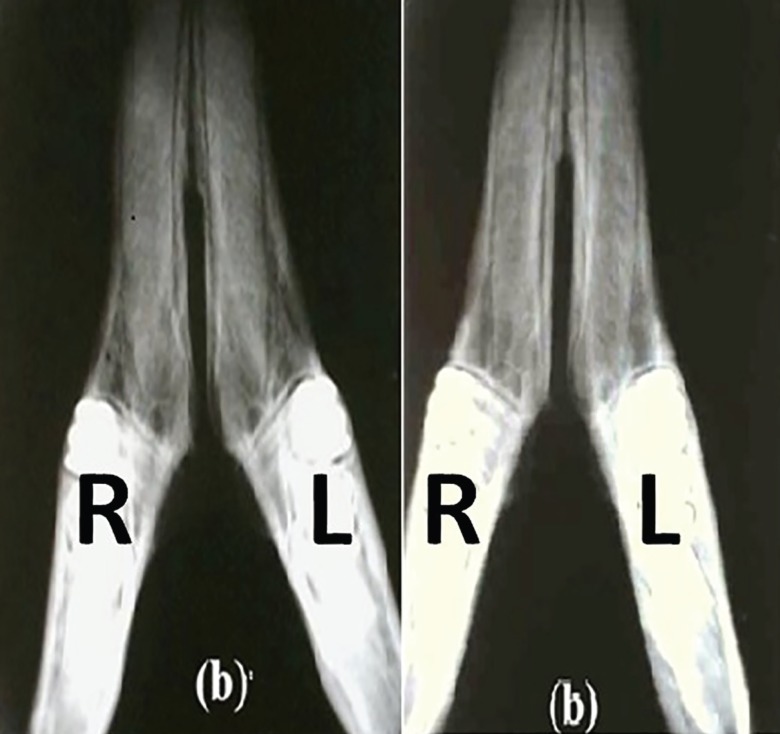
Radiographic observation on day of injury. **a)** Left R: the defect filled with fibrin glue; Left L: the control defect, **b)** Right R: the defect filled with fibrin glue; Right L: the defect filled with autologous bone graft after 4 weeks.

**Fig. 5:**
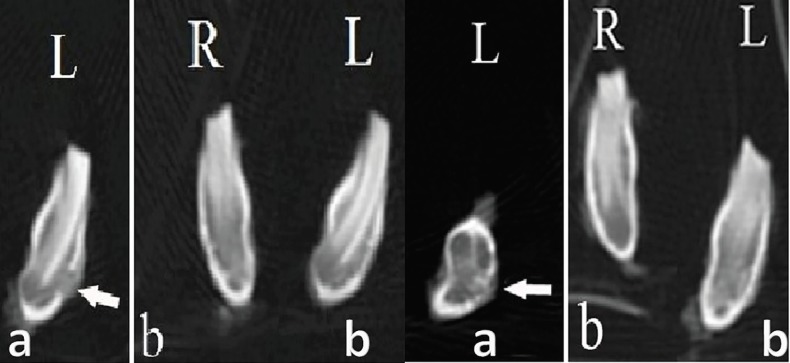
Coronal CT scanning observation after 4 weeks. Left aL: the control defect, Left bR: the defect filled with fibrin glue; Left bL: the defect filled with autologous bone graft. Coronal CT scanning observation 8 weeks after surgery. Right aL: the control defect, Right bR: the defect filled with fibrin glue; Right bL: the defect filled with autologous bone graft.

Radiography denoted to formation of cortical bone in treatment groups. In control group, no bony reconstruction was seen at 4^th^ weeks post-surgery (Figure 4). After 8 weeks, new cortical bone formation without any integration was visible (Figure 4). Coronal CT scanning showed reconstruction of cortical bone in treatment groups. In control defects, no bony reconstruction was seen at 4^th^ weeks post-surgery (Figure 5). After 8 weeks, new cortical bone formation without any integration was noted (Figure 5).

**Fig. 6:**
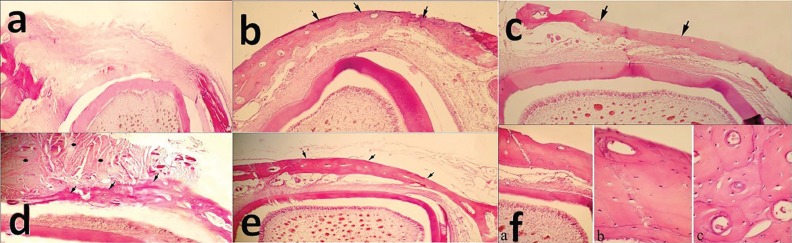
Histopathological evaluation of the bone samples 4 weeks after surgery: a) the control defect; x60), b) the defect was filled by fibrin glue alone; x60, c) the defect was filled by autologous bone graft; x150. At the 8^th^ week post-surgery: d) formation of a thin cortical bone bridge in control group, while ﬁbrous-immature bone tissue is still presence; x60, e) A complete cortical bone formation that is thicker than 4 weeks post-treatment in fibrin glue group; x60, f) Lamellar bone with less Haversian system in autologous bone graft group; f-a) x150, f-b) x600, f-c) x600 (H&E).

At 4 weeks post operation, in the defects filled with fibrin glue, new cortical bony bridge formation was seen in three out of four cases. Neither foreign body reaction nor inflammatory response was visible. In one out of four cases of the defects filled with autologous bone graft, newly formed bony bridge with active osteoblasts could be recognized while presence of fibrous tissue with mononuclear cells around remained bony particles were visible. In other defects of this group, a thin cortical bony bridge formation was seen (Figure 6).

In the control group at 8^th^ weeks post-surgery, a thin cortical bony bridge was detected while ﬁbrous-immaure bony tissue was also present (Figure 6). In all of the treatment groups, cortical bony bridge was well formed and had more thickness in comparison to those after 4 weeks. However, Haversian system was less than mature lamellar cortical bone. The data of the quantitative scores according to the Emery’s *et al.* and Friedman grading system were summarized in tables 1 and 2, respectively. According to the Emery’s *et al.* scoring method, at 4^th^ week post-surgery, there was a statistical difference between the fibrin glue group and the control group as well as the autologous bone graft group with the control group (*p*=0.008). 

**Table 1:** Histopathological scoring results according to Emery’s et al. system.

**Time **	**Groups**	**Mean±SD**	***p*** **value**
4 weeks	empty defect^abcd^	1.40±1.51	0.008
	fibrin glue^ba^	6.50±1.00	
	autologous bone graft^da^	6.75±0.50	
8 weeks	empty defect^ef^	5.6±0.89	0.02
	fibrin glue^fe^	7	
	autologous bone graft^h^	6.67±0.33	

SD, Standard Deviation, Dissimilar letters indicate significant differences using Mann–Whitney U test (*p*<0.05).

**Table 2:** Histopathological scoring results according to Ann and Friedman system.

**Time **	**Groups**	**Mean±SD**	***p*** ** value**
4 weeks	empty defect^abc^	0	0.019
	fibrin glue^ba^	2.5±1.00	
	autologous bone graft^d^	1.50±1.732	
8 weeks	empty defect^e^	2.20±0.83	0.09
	fibrin glue^f^	3	
	autologous bone graft^h^	2.67±0.57	

SD, Standard Deviation, Dissimilar letters indicate significant differences using Mann–Whitney U test (*p*<0.05).

In contrast, statistical analysis showed no significant differences among the other groups (*p*>0.05). After 8 weeks, there were significant differences between the fibrin glue group and the control group (*p*=0.028). No statistically significant differences were found among the other groups (*p*>0.05). Based on Friedman grading system, at 4^th^ week post-surgery, there was a statistical difference in fibrin glue group and the control group (*p*=0.006). There was no statistically significant difference among the other groups (*p*>0.05). Statistical analysis showed no significant differences among the groups at 8^th^ week post-surgery (*p*>0.05).

The average and standard deviation of the thickness of the newly formed cortical bone in different groups are summarized in Table 3. After 4 weeks, significantly more bone thickness was detected in treatment groups in comparison to the control group (*p*=0.003). No statistically significant difference was found between the fibrin glue and the autologous bone graft group (*p*>0.05). At 8^th^ weeks post-surgery, there was a statistical difference in bone thickness between the treatment group and the control group (*p*=0.006). In contrast, statistical analysis showed no significant differences among the other groups (*p*>0.05).

**Table 3:** Histopathological scoring results according to new cortical bone thickness.

**Time **	**Groups**	**Mean±SD** **(microns)**	***p*** ** value**
4 weeks	empty defect^abcd^	0	0.003
	fibrin glue^bac^	156±51.15	
	autologous bone graft^dac^	193.5±70.82	
8 weeks	empty defect^efgh^	127.8±27.08	0.006
	fibrin glue^fe^	263±27.17	
	autologous bone graft^he^	342±15	

SD, Standard Deviation, Dissimilar letters indicate significant differences using Mann–Whitney U test (*p*<0.05).

## DISCUSSION

Rabbit was previously shown to be a suitable animal model in human regenerative medicine.^17^ However, in rabbits; creation of discontinuity and any defect in the mandible, because of limited surgical access is difficult, but there is a relatively large diastema between incisor and first premolar teeth, so it can be an appropriate site for experiments on the mandible.^18^ Osteogenesis, osteoconduction or osteoinduction as three important characteristics of an ideal graft material, that in autologous bone graft have been regarded as gold standard in restoration of bony defects even it may be accompanied by some drawbacks.^19^

In this study, autologous fibrin glue was prepared by using Thorn *et al.*^14^ scoring method containing large amounts of platelets and fibrinogen. The study before ^20^ demonstrated that fibrin can be used as a good bead to restore bony defects due to positive effect of periosteal cells on fibrin beads in reconstruction of bony defects of rabbit after 28 days. An experimental study in mouse model showed the positive role of fibrin glue as potential osteoinductive biologic glue and as an important factor to improve early differentiation of osteogenic cells.^21^

The study done by Lee *et al*.^22^ on repairing of maxillary sinus faults in dog showed that simultaneous application of autologous bone graft and platelet enriched fibrin glue resulted to significantly greater bone formation than group treated with autologous bone graft alone. Also, osseous integration in group treated with fibrin glue was greater than the group which was treated by autologous bone graft. Computed tomography and histomorphometric analysis of the work done previously^23^ showed positive effect of fibrin glue alone or in combination with hydroxyapatite β- tricalcium phosphate on repair of calvarial bone defect in rabbit at 4^th^ and 8^th^ weeks post-surgery.

Early and accurate diagnoses are the main factors in curing of maxillofacial fractures, while computed tomography enrolls the diagnosis. Additionally, in comparison to radiography, this method can provide higher contrast resolution.^24^^,^^25^ In the present study, CT scanning and histopathological surveys showed that fibrin glue scaffold and autologous bone graft (regardless to bone graft problems) had appropriate osteogenesis in comparison with the control group. Using of fibrin glue in mandibular defect reconstruction seems to be usefulness because it can be easily located and shaped.^14^

Works done before ^25^^,26^ showed unlike collagen and xenogenic gelatin which may result to inflammatory responses, risk of foreign body reaction or infection in using autologous fibrin glue reduced. In our study, gel like fibrin glue was produced after combination of thrombin and fibrinogen which was easily applied to the defect. In histopathological evaluation, in groups treated with fibrin glue, glue or foreign body reaction was absent showing its biocompatibility and biodegradability. The comparative results of histopathological evaluations in treated and control groups revealed that healing process in fibrin glue scaffold was better than other groups with a significant (*p*<0.05) increase in the thickness of new cortical bone at 4^th^ week. 

Our findings demonstrated that application of fibrin glue can be suggested as a good bony graft substitute that is non-invasive in comparison to autologous bone graft for reconstruction of maxillofacial bony defects.
